# Risk factors and poor prognostic factors of preeclampsia in Ibn Rochd University Hospital of Casablanca: about 401 preeclamptic cases

**DOI:** 10.11604/pamj.2018.31.225.14401

**Published:** 2018-12-06

**Authors:** Meriem Benfateh, Souadou Cissoko, Houssine Boufettal, Jean-Jacques Feige, Naima Samouh, Touria Aboussaouira, Mohamed Benharouga, Nadia Alfaidy

**Affiliations:** 1 Unité de Culture Cellulaire, CED, Faculté de Médecine et de Pharmacie, Hassan II University of Casablanca, Maroc; 2 Service C de Gynécologie, CHU Ibn Rochd, Faculté de Médecine et de Pharmacie, Hassan II University of Casablanca, Maroc; 3 Institut de Biosciences et Biotechnologie du CEA de Grenoble, Unité U1036 INSERM, Grenoble, France; 4 Commissariat à l’Energie Atomique (CEA), DSV-iRTSV, 17 rue des Martyrs, France-Université Grenoble-Alpes, Centre National de la Recherche Scientifique, UMR 5249, 38054 Grenoble Cedex 9, France

**Keywords:** Preeclampsia, risk factors, maternal prognostic, fetal prognostic

## Abstract

Preeclampsia is a gestational pathology that complicates 2 to 8% of pregnancies and is one of the major causes of maternal and fetal morbidity and mortality worldwide. The aim of this work was to study the epidemiological profile of preeclampsia in Casablanca and to identify risk factors as well as factors of poor maternal and fetal prognosis. 401 preeclamptic cases were collected in the gynecology-obstetrics “C” Service of Lalla Meryem Maternity of Ibn Rochd University Hospital of Casablanca (2010-2011) were included in this study and a statistical analysis with the SPSS software version (16.0) was performed. We used the Chi-2 test to analyze qualitative variables and Student's test and ANOVA (analysis of variance) for quantitative variables. The incidence of preeclampsia was (7.1%). The epidemiological profile was that of a primipara (57.6%), average age 30 years and (66.8%) of pregnancies were not followed. Multiparity was a factor of poor maternal prognosis (p = 0.007). The low gestational age and no prenatal care were factors of maternal as well as fetal prognosis. Risk factors frequently found in our patients were obesity (15.21%) and chronic hypertension (5.73%) as vascular-renal history; abortion (16.46%) and perinatal death (5.24%) as obstetric history. Preeclampsia is a common obstetric pathology in our context. Better prenatal care and early diagnosis could reduce its incidence.

## Introduction

Preeclampsia is a gestational pathology, defined as the association of pregnancy-induced hypertension (BP≥140/90mmHg) and proteinuria of (≥300mg/24h) after 20 weeks of gestation [1]. It affects approximately 0.5 to 7% of pregnancies [2] and is a leading cause of maternal and fetal morbidity and mortality worldwide [3]. This rate is higher in low socioeconomic status and areas with high prevalence of cardiovascular diseases [4]. Several risk factors have been proposed to characterize women at high risk of preeclampsia, including nulliparity, older age, chronic hypertension, and pregnancy diabetes mellitus [5]. Moreover, patients with preeclampsia are at increased risk of developing cardiovascular disease, renal and neurological disorders later in life which determines the risk profile of the child' future health [6]. It is a complication of pregnancy, which also has long-term effects on maternal health [7] and is one of the major causes of premature delivery usually medically indicated for the benefit of the mother in developed countries, which leads to infant morbidity and substantial excess health care expenditure [8]. Since, the mechanisms of diseases onset remains unclear and currently, no therapeutic approaches are available to either treat or prevent preeclampsia, which just still based on the prevention of the malignant hypertension and ending the pregnancy [1]. Furthermore, there is no real predictive factors of this pathology [9]. However, the identification of prognostic factors of pregnant women at risk of preeclampsia could be of great importance so as to improve the prognosis of our patients and their childbirths. Incidence of preeclampsia throughout the world varies according to the authors, the studied population and the definition used. It also differs between different ethnic groups, from one country to another and even from one region to another within the same country. In the US, the frequency had increased from 3.4% to 3.8% over the last 30 years [10]. In France, the incidence of preeclampsia was estimated to 1-3% in nulliparous patients and between 0.5 to 1.5% in multiparous patients [11]. In Spain, the frequency was 1 to 2% [4]. In Anglo-Saxon countries, the incidence ranges from 3 to 7% in primiparae and 1 to 3% in multiparous [3]. In black Africa, the prevalence of preeclampsia was of around 25% [12]. Frequency of preeclampsia was found to be 44% in sub-Saharan Africa [13]. In a Senegalese study, a frequency of 14.9% was found in relation to all the hypertensive states associated with pregnancy [14]. In Morocco, the exact epidemiological situation of preeclampsia remains little investigated. The aim of this work is to evaluate the epidemiological profile of preeclampsia in Casablanca and to identify risk factors as well as factors of poor maternal and fetal prognosis factors by a retrospective study including 401 preeclamptic patients.

## Methods

Retrospective study was conducted during the two-year period from the first January 2010 to 31 December 2011, at the Department of Gynecology-Obstetrics “C” Service of Lalla Meryem Maternity of Ibn Rochd University Hospital of Casablanca. Included patients were those having preeclampsia defined by hypertension (BP≥140/90mmHG) associated with significant proteinuria (≥0.3g/24h or ≥2 crosses with labstix ^®^) with or without edema and/or patients with one or more complications occurring in the context of preeclampsia. Out of 5671 births were reported during this period, a total of 660 cases of all the records of hypertension associated with pregnancy was collected, out of which 401 cases had preeclampsia. The data information were abstracted from the medical record and supplemented with data collected specifically for the study, that were processed by a statistical study using the Statistical Software Package for Social Science (SPSS) software version (16.0). A univariate analysis was performed in order to reveal the characteristics of the population studied. Chosen parameters were: frequency, maternal age, gestational age, (medical, obstetric, gynecological and surgical) history, parity and monitoring of pregnancy. A bivariate analysis was performed to identify factors of poor maternal prognosis. The occurrence of complications (maternal or fetal accident) was considered as the variable to be explained and the other variables as explanatory variables. We used the Chi-2 square test to analyze the qualitative variables and Student and Anova tests (analysis of variances) for the quantitative variables. The significance level was set at 5% (p <0.05: was considered statistically significant). Ethics: informed consent approved by the University Hassan II comity of ethics, was obtained from each patient included in this study.

## Results

### Univariate analysis

**Frequency:** out of 5671 births registered, 660 cases of hypertension associated with pregnancy are recorded, out of which 401 cases have preeclampsia (Table 1). Preeclampsia (either pure or added) represents 60.76% of HTAs encountered in our department during the study period, with a frequency of 7.07%.

**Table 1:** types of preeclampsia according to HTA among the 401 preeclamptic patients studied in the Gynecology-Obstetrics “C” Service of Lalla Meryem Maternity of Ibn Rochd University Hospital of Casablanca (2010-2011)

Preeclampsia types	Number of cases	Percentage (%)	Frequency (%)
Preeclampsia	378	94.26	6.66
Added preeclampsia	23	5.74	0.41
TOTAL	401	100	7.07

**Maternal age:** the average age of preeclampsia patients is 30 years with extremes ranging from 15 to 47 years. The range of age group most affected is between 24 and 34 years old (47.38%), followed by the age group of 35 to 47 years (31.92%). Patients aged 15 to 23 years are the least affected (20.70%).

**Gestational age:** the average gestational age on admission found to be 36.2 weeks of gestation with extremes ranging from 24 to 34 SA. More than two thirds of the patients (70.82%) have a gestational age (≥ 36 weeks of gestation) (fetal maturity assumed to be acquired); whereas 27.19% are not at term of which 9.23% are far of the term with a gestational age (<32 weeks of gestation) (risk of severe prematurity). For 1.99% of preeclampsia patients, gestational age is not specified.

**Medical history:** of the 401 preeclampsia patients included in this study, 74 (18.45%) have a medical history (Table 2). Obesity is the most frequent risk factor in our preeclamptic patients (15.21% of cases) followed by chronic hypertension (5.73% of cases). Some patients have multiple medical history at same time.

**Table 2:** medical history among the 401 preeclamptic patients studied in the Gynecology-Obstetrics “C” at the Service of Lalla Meryem Maternity of Ibn Rochd University Hospital of Casablanca (2010-2011)

Medical history	Number of cases	Frequency %
Obesity	61	15.21
Chronic HTA	23	5.73
Diabetes	11	2.74
Cardiopathy	5	1.25
Nephropathy	3	0.75
Urinary tract infection	3	0.75
Asthma	3	0.75
Lupus	1	0.25
Behçet’s disease	1	0.25
No history	290	72.32
TOTAL	**401**	**100**

**Obstetric history:** of the 401 preeclampsia patients included in this study, 105 (26.18%) have an obstetric history (Table 3). Abortion is the most frequent antecedent in (16.46% of cases). Some patients have several obstetric antecedents at the same time.

**Table 3:** obstetric history among the 401 preeclamptic patients studied in the Gynecology-Obstetrics “C” at the Service of Lalla Meryem Maternity of Ibn Rochd University Hospital of Casablanca (2010-2011)

Obstétrical history	Number of cases	Frequency %
Abortion	66	16.46
Intra uterine fetal death	21	5.24
Neonatal death	22	5.48
Caesarean section	32	7.98
Preeclampsia	20	4.98
Eclampsia	2	0.50
Retro-placental hematoma	2	0.50
Ectopic pregnancy	3	0.75
No history	233	58.11
TOTAL	168	100

**Gynecological history:** only 9 patients have gynecological history in (2.24% of cases), 5 cases with uterine fibroid, 2 cases with ovarian cyst, 1 case with breast nodule and 1 case with uterus bicervical.

**Surgical history:** surgical history is not reported in 8 patients (2%), with 5 cases with cholecystectomy, 1 case with appendectomy, 1 case with intestinal occlusion and 1 case with hydatid cyst of the liver.

**Parity:** preeclamptic patients are classified into three groups according to parity: primiparae (one delivery after 28 weeks), biparae (two to four deliveries) and multiparae (≥ 5 deliveries). More than half of our patients are primiparae (57.61%), 1.7 and 6.8 times more numerous than biparae (33.91%) and multiparae (8.48%) respectively.

**Pregnancy monitoring:** in this study, 133 patients are regularly monitored on the obstetrical level in (33.17% of cases), while two-thirds (268 cases) are not followed (66.83% of cases).

### Bivariate analysis

**Factors of poor maternal prognosis:** maternal age, gestational age, parity and non-prenatal follow-up are reported as factors of poor maternal prognosis in our study (Table 4).

**Table 4:** epidemiological factors of poor maternal prognosis among the 401 preeclamptic patients studied in the Gynecology-Obstetrics “C” Service of Lalla Meryem Maternity of Ibn Rochd University Hospital of Casablanca (2010-2011)

Epidemiological prognostic factors	Associated complications	P
Maternel age < 24 years	Eclampsia	p = 0.02
Maternel age> 34 years	Acute rénale impairment Acute lung edema	p = 0.015 p = 0.026
Multiple	HELLP syndrome	p = 0.007
Low gestational age < 34 weeks of amenorrhea	Eclampsia	p = 0.0001
	HELLP syndrome	p < 0.0001
	Retro-placental hematoma	p = 0.0022
	Acute renal failure	p = 0.026
	Maternal death	p = 0.049
Non-prénatal follow-up	HELLP syndrome	p = 0.006
	Eclampsia	p = 0.03
	Maternal death	p = 0.044

**Factors of poor fetal prognosis:** poor fetal prognosis factors found in our study are the advanced maternal age and low gestational age (Table 5).

**Table 5:** epidemiological factors of poor fetal prognosis among the 401 preeclamptic patients studied in the Gynecology-Obstetrics “C” at the Service of Lalla Meryem Maternity of Ibn Rochd University Hospital of Casablanca (2010-2011)

Epidemiological prognostic factors associated complications	Associated complications	p
Maternel age> 34 years	Prematurity	p = 0.04
Early gestational age< 34 weeks of amenorrhea	Fetal hypotrophy	p = 0.0004
	Prematurity	p < 0.0001
	Neonatal suffering	p < 0.0001
	Neonatal respiratory distress	p = 0.0002
	perinatal death	p < 0.0001

## Discussion

The frequency of preeclampsia in Casablanca is 7.07%. Moroccans studies noted a variable rate between frequencies found in other cities ranging from 50 to 73% [15]. Therefore, this rate was 44% in sub-Saharan countries 44% [13], compared with developed countries that reported a frequency of 0.5-7% [3, 10, 11]. In our study, the mean maternal age of our preeclamptic patients are 30 years. This trend can be explained by the increased frequency of marriage and early pregnancies in this age group in our context. Young maternal age between 25 and 35 years has been reported to be as a risk factor for preeclampsia in several studies [12]. According to various authors [8], is the advanced maternal age that constitutes a factor of poor maternal prognosis. Numerous studies have reported a risk of preeclampsia multiplied by 2 to 4 for women over 35 years of age [11]. In our study, preeclamptic patients age under than 24 years of age are significantly more at risk of eclampsia, while those aged > 34 years are significantly more at risk for acute renal failure, acute edema of the lung. The frequency of prematurity in our study is significantly higher in our preeclampsic patients over 34 years of age compared to those aged 24 to 34 years and those aged less than 24 years. Thus, we suggest that the advanced maternal age is a factor of poor prognosis in our study. The development of preeclampsia at an early gestational age (<34 weeks of gestation) have been associated with significant maternal and perinatal morbidity and mortality [6]. In our study, preeclampsia patients with a gestational age (<34 weeks of gestation) has worse prognosis compared to those patient with a gestational age (≥34 weeks of gestation), which is often accompanied by complications such as (HELLP Syndrome, eclampsia, retroplacental hematoma, acute renal failure and maternal death). The results show a significantly higher frequency of fetal hypotrophy, prematurity, neonatal distress, neonatal respiratory distress and perinatal death in neonates of patients with a gestational age (<34 weeks of gestation) compared with those of preeclamptic patients having a gestational age (≥ 34 weeks of gestation). Similarly, a recent work has reported the development of preeclampsia at an early gestational age (<34 weeks of gestation) [3]. Thus, early gestational age (<34 weeks of gestation) may be considered as a factor of poor maternal and fetal prognosis. Preeclampsia complicates 2 to 8% of pregnancies worldwide [6]. Much research reported that primigesity and primiparity predispose to preeclampsia: the proportion of preeclampsia is high in primigravida and primipara women, regardless of their age [16]. The incidence of preeclampsia was more frequent in nulliparous than primiparous and multiparous [17]. In our study primiparous as a maternal prognosis are the most exposed group. Similar results have been reported primiparity as a risk factor of preeclampsia and its proportion was variable in the literature [18]. Therefore, other risk factors found in the literature include the advanced maternal age and multiparity [3]. In our study, multiparous preeclampsia patients have more complications compared to primiparous, with a significantly higher frequency of HELLP syndrome. These results support the fact that multiparity is a factor of poor maternal prognosis. In our study, preeclampsia patients present a medical history with high frequency of vasculo-renal antecedents. These antecedents (obesity, hypertension, diabetes, nephropathy, cardiopathy) increase the risk of preeclampsia by the vascular lesions that they determine [8]. Obesity is the most common risk factor, followed by chronic hypertension and diabetes in our study. These three factors have often been presented as a risk factors for pre-eclampsia [19]. In women with pre-gestational diabetes, preeclampsia complicates 10% to 20% of pregnancies, three times more than reported in normal pregnancies with higher risk of preeclampsia in patients with type 1 diabetes and pregestational microalbuminuria [20]. Obesity confers a high risk of fetal and maternal consequences of preeclampsia [21]. In our study, pre-eclampsia patients have an obstetric history in order of frequency: abortion, perinatal death, pre-eclampsia, eclampsia and/or retroplacental hematoma. There is no significant difference in maternal or fetal complications in patients with or without obstetric history. However, gynecological and surgical history are less present in preeclampsia patients. In our study, preeclampsia patients having no obstetric follow-up have a worse prognosis compared to those whose pregnancy was followed, with a significantly higher rate of HELLP syndrome, Eclampsia and maternal death, which is similar to the results found in some other studies that have reported the strong association of the complication of HELLP syndrome with the increased risk of maternal morbidity and mortality [22]. Thus, the non-prenatal follow-up represent a factor of poor maternal prognosis in our study.

## Conclusion

Preeclampsia is still a public health problem due to its high incidence and the presence of many severe cases in our context. It is one of the main causes of maternal and fetal morbidity and mortality. In this study, obesity and chronic hypertension were the most frequent risk factors as a vasculo-renal history, followed by abortion and perinatal death as obstetric history. In this context, only earlier diagnosis and closer monitoring could improve the prognosis of our preeclamptic patients in order to reduce its incidence.

### What is known about this topic

- The epidemiological's situation of preeclampsia have already been reported in several research in Europe and North America;
- Daily hospital practice in various maternity hospitals through Morocco seems to report a high frequency of preeclampsia compared to the literature;
- No work that has yet drawn up the epidemiological profile of this pathology in Morocco.

### What this study adds

- This work is an original study since it reports, via a statistical study by SPSS software, the epidemiological profile and the various risk factors of preeclampsia in this population and the poor prognosis factors related to this pathology.

## Competing interests

The authors declare no competing interests.
